# *Scrophularia koraiensis* Nakai Attenuates Allergic Airway Inflammation via Suppression of NF-κB and Enhancement of Nrf2/HO-1 Signaling

**DOI:** 10.3390/antiox9020099

**Published:** 2020-01-24

**Authors:** Tae-Yang Jung, A Yeong Lee, Jun-Ho Song, Min Young Lee, Je-Oh Lim, Se-Jin Lee, Je-Won Ko, Na-Rae Shin, Jong-Choon Kim, In-Sik Shin, Joong-Sun Kim

**Affiliations:** 1 College of Veterinary Medicine (BK21 Plus Project Team), Chonnam National University, 77 Yongbong-ro, Buk-gu, Gwangju 61186, Korea; jupiterriot@naver.com (T.-Y.J.); dvmljo@gmail.com (J.-O.L.); xhdhksdl123@naver.com (S.-J.L.); rheoda@gmail.com (J.-W.K.); tlsskfo870220@gmail.com (N.-R.S.); toxkim@jnu.ac.kr (J.-C.K.); 2 Herbal Medicine Resources Research Center, Korea Institute of Oriental Medicine, 111 Geonjae-ro, Naju-si, Jeollanam-do 58245, Korea; lay7709@kiom.re.kr (A.Y.L.); songjh@kiom.re.kr (J.-H.S.); 3 College of Pharmacy, Research Institute of Pharmaceutical Sciences, Kyungpook National Univeristy, 80 Daehak-ro, Buk-gu, Daegu 41566, Korea; vetmedic@knu.ac.kr

**Keywords:** *Scrophularia koraiensis*, Asthma, Nrf2, HO-1, NF-κB

## Abstract

*Scrophularia koraiensis* Nakai (Scrophulariaceae) is a medicinal herb that grows in Korea and which has been widely used to treat fever, edema, neuritis and laryngitis. Hence, we evaluated the anti-inflammatory and antioxidant effects of the ethanol extract (SKE) of *S. koraiensis* Nakai in an ovalbumin (OVA)-induced mouse model. We injected 20 μg of OVA with 2 mg of aluminum on day 0 and day 14 to induce allergic airway inflammation in six-week-old BALB/c mice, and mice were challenged with 1% OVA by nebulization for 1 h on days 21, 22, and 23. SKE was orally administered at 20 mg/kg and 40 mg/kg from day 18 to 23, and its effects were compared with those of montelukast treatment. SKE significantly reduced proinflammatory cytokines, inflammatory cell counts, immunoglobulin-E, and airway hyperresponsiveness during the OVA-induced allergic airway inflammation model; it also reduced airway inflammation and mucus production. In addition, SKE reduced the OVA-induced nuclear factor kappa B (NF-κB) phosphorylation in lung tissues while enhancing nuclear factor erythroid-derived 2-related factor (Nrf-2) and heme oxygenase-1 (HO-1) expression. In conclusion, SKE showed the protective effects on OVA-induced allergic airway inflammation via the suppression of NF-κB phosphorylation and the enhancement of the Nrf2/HO-1 signaling pathway. These results indicate that SKE is a potential therapeutic agent for allergic airway inflammation.

## 1. Introduction

Asthma is a chronic inflammatory disease that affects 275 million people worldwide [1]. It is caused by exposure to specific allergens that aggravate inflammation, mucus hypersecretion, and airway hyperresponsiveness (AHR), resulting in the loss of normal lung function [2,3]. The clinical signs of asthma are wheezing, dyspnea, chest tightness, and cough due to limitations of airflow to the respiratory tracts [4]. The development of asthma is associated with the increased production of pro-inflammatory cytokines, chemokines, growth factors, and reactive oxygen species (ROS), which eventually lead to eosinophilia, AHR, and mucus overproduction [5,6,7]. There is an ongoing search for an effective asthma treatment that targets these varied pathways [8,9,10].

Oxidative stress is associated with the development of asthma [3]. Oxidative stress occurs because of an imbalance between external oxidation inducers and the antioxidant response within cells, resulting in the production of ROS [11], which induces asthmatic responses via the activation of inflammatory signaling [12,13]. The antioxidant signaling molecules, nuclear factor erythroid-derived 2-related factor 2 (Nrf2) and heme oxygenase-1 (HO-1), have been targeted by many researchers to counter excessive ROS during asthma development [14]. HO-1 is an antioxidant enzyme that is involved in oxidative stress, as well as in cell damage and inflammatory disease [15]. The expression of HO-1 inhibits the expression of nuclear factor kappa B (NF-κB), thereby reducing the expression of inducible nitric oxide synthase (iNOS), resulting in an alteration in cytokine gene expression [16]. Therefore, HO-1 may exert anti-asthmatic effects by decreasing ROS production and inflammatory response.

The genus *Scrophularia* L., which belongs to the family Scrophulariaceae, consists of about 200 species. This genus is widely distributed across the temperate regions of the Northern Hemisphere. Moreover, various species of the genus *Scrophularia* have been used as therapeutic agents for fever, edema, constipation, neuritis and laryngitis [17,18]. One of these, *Scrophularia koraiensis* Nakai, is a rare medicinal herb that grows in Korea and has been used as an antipyretic and anti-inflammatory agent in the past [19]. The pharmacological properties of *S. koraiensis* have been described, but the effect of *S. koraiensis* on various diseases has not been studied. In the present study, we investigate the effects of *S. koraiensis* on ovalbumin-induced allergic airway inflammation, focusing on its anti-inflammatory and antioxidant properties.

## 2. Materials and Methods

### 2.1. Animals

Female-specific pathogen-free 6-week-old BALB/c mice were purchased from SAMTAKO (Osan, Korea). The mice were maintained under standard conditions (on a 12 h night/day cycle, at a humidity of 55 ± 5 % and temperature of 22 ± 2 °C) and fed ad libitum. All experiments were conducted according to a protocol approved by the Chonnam National University Institutional Animal Care and Use Committee. (CNU IACUC-YBR-2016-19, Gwangju, Korea)

### 2.2. Materials and Instruments

*Scrophularia koraiensis* Nakai was collected from the experimental field of the National Institute of Horticultural and Herbal Science (NIHHS, Chungcheongbuk-do, Korea, 36°56’24.7” N, 127°44’56.1” E), and medicinal parts and plant voucher specimens (2–18–0145, KIOM-2019-54) were deposited in the Korean Herbarium of Standard Herbal Resources (Index Herbariorum code: KIOM) at the Korea Institute of Oriental Medicine, Naju, Korea*. S. koraiensis* is distinguished from closely related species, *Scrophularia buergeriana* Miq. (Table S1) because it has an acuminate apex, regular double serrated margins, obtuse base, and acuminate calyx lobes (Figure S1). *S. koraiensis* (69.15 g) was refluxed in 70% ethanol (*v/v*) for 2 h, and the was extract filtered and evaporated in vacuo. The yield of the 70% ethanol extract of *S. koraiensis* (SKE) was 43.96% (*w/w*) at 4 °C.

Aucubin, harpagide, and harpagoside were purchased from Shanghai Sunny Biotech (Shanghai, China), and 8-acetyl harpagide and angoroside C were obtained from ChemFaces (Wuhan, Hubei, China). High performance liquid chromatography (HPLC) grade water, methanol, and acetonitrile were from Merck (Darmstadt, Germany). The HPLC system (Waters Corporation, Milford, MA, USA) was assembled by using the Waters e2695 Separation Module, a 2998 PDA detector, an Acquity QDa detector, and a micro-splitter (IDEX Health & Science LLC, Oak Harbor. WA, USA).

### 2.3. HPLC analysis

SKE (110.5 mg) was dissolved in 10 mL of 70% ethanol and filtered through a 0.2 μm syringe filter. The column was a Kinetex Biphenyl 100A (4.6 × 250 mm, 5 μm, Phenomenex Inc., Torrance, CA, USA). The mobile phase was mixed with 0.05% aqueous formic acid (A), acetonitrile (B), and methanol (C), and a gradient program from 100% A (2 min) → 96% A (3% B and 1% C, 7 min) → isocratic 85% A (11% B and 4% C, from 15 min to 20 min) → 70% A (23% B and 7% C, 35 min) → 50% A (35% B and 15% C, 45 min) → 30% A (50% B and 20% C, 55 min) was run. The flow rate was 0.8 mL/min, and the injected volume was 10 μL. The UV wavelength was monitored from 195 to 400 nm. The QDa detector was set as follows: a nitrogen carrier gas, an electrospray interface (ESI) capillary of 0.8 kV, a 600 °C probe temperature, a 15 V con voltage, a 120 °C source temperature, and a 20:1 split. The mass range was from 300 to 850 *m/z*. The peaks were confirmed by using the standard compound peaks observed in the UV and MS spectra. The sample peaks were identified by comparing the retention time and molecular weight of each sample with that of the standard compound peaks.

### 2.4. OVA-Induced Allergic Airway Inflammation Model

**Figure 1:**
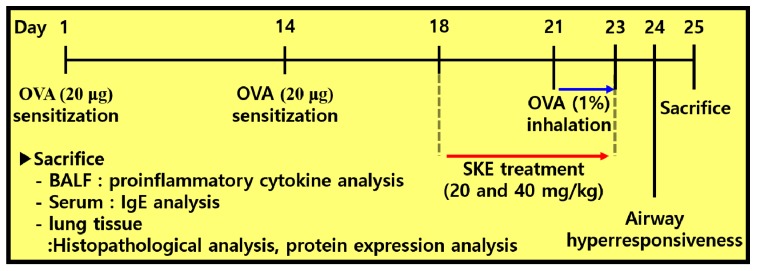
The experimental procedure.

To induce allergic airway inflammation in mice, intraperitoneal injections of OVA (20 μg, Sigma-Aldrich, St. Louis, MO, USA) that was mixed with 2 mg of aluminum hydroxide (Sigma-Aldrich) were administered on days 1 and 14. Subsequently, mice received an OVA challenge (1% *w/v* in phosphate buffered saline (PBS)) for 1 h from days 21 to 23. Montelukast (10 mg/kg) and SKE (20 and 40 mg/kg) were administered by oral gavage from days 18 to 23. On day 24, AHR was evaluated by using whole-body plethysmography (OCP3000 instrument, Allmedicus, Seoul, Korea). AHR was evaluated following methylcholine inhalation (0, 10, 20 and 30 mg/mL in PBS) for 3 min. The results of AHR were expressed as a dimensionless parameter, enhanced pause (Penh). The mice were divided into 5 groups (*n* = 5); NC (normal control; PBS sensitization, PBS challenge and PBS administration), OVA (OVA sensitization, OVA challenge and PBS administration), Mon (OVA sensitization, OVA challenge, and montelukast administration), SKE 20 and 40 (OVA sensitization, OVA challenge, and SKE administration (20 and 40 mg/kg, respectively). The experimental procedure is shown in Figure 1.

### 2.5. Measurement of Allergic Parameters in Bronchoalveolar Lavage Fluid (BALF) and Serum

On day 25, mice were anesthetized with alfaxalone (Jurox, Rutherford, Australia), and blood samples were collected from the cauda vena cava. The blood samples were centrifuged for 20 min at 200 g to separate the serum. The total immunoglobulin E (IgE) and OVA-specific IgE were measured by an enzyme linked immunosorbent assay (ELISA) (BioLegend Inc., San Diego, CA, USA). To collect the bronchoalveolar lavage fluid (BALF), we performed tracheostomy in the mice and inserted endotracheal tubes. PBS (0.7 mL) was injected into the lung and removed via the tube, and the process was repeated once. The collected BALF was centrifuged (200 g, 4 °C, 10 min). The supernatants were collected in new tubes for measuring pro-inflammatory cytokines interleukin (IL)-5 and IL-13 by using ELISA. The remaining pellet was dissolved in 200 μL of PBS, followed by centrifugation through Cytospin (Hanil Electric, Wonju, Korea) to attach inflammatory cells to the slides. Slides were stained by a Diff-Quik reagent (Sysmex, Kobe, Japan) for counting the different cells in the BALF.

### 2.6. Histopathology of Lung Tissue

To perform histological examinations, the left lung was fixed in formalin after BALF sampling. The lung tissue samples were embedded in paraffin blocks, and 4 μm sections were prepared. Tissues were stained in hematoxylin and eosin (H and E, Sigma-Aldrich) and periodic acid-Schiff (PAS, IMEB Inc., San Marcos, CA, USA) to evaluate airway inflammatory responses and mucus production, respectively. A quantitative analysis of inflammation and mucus production in lung tissue was performed with the use of an image analyzer (IMT i-Solution software, IMT i-Solution Inc., Vancouver, BC, Canada).

Immunohistochemistry (ICH) was performed to evaluate inducible nitric oxide synthase (iNOS) and HO-1 expression in lung tissue by using a commercial kit (Vector Laboratories, Burlingame, CA, USA). The anti-mouse iNOS antibody (diluted 1:200, Abcam, Cambridge, UK) and anti-mouse HO-1 (diluted 1:200, Abcam) were used as primary antibodies. The slides were examined under a light microscope (Leica, Wetzlar, Germany) in a completely blinded manner.

### 2.7. Western Blotting

To identify the proteins and signaling pathways within the lung tissue, the right lung was homogenized with a tissue lysis/extraction reagent (Sigma-Aldrich). The total protein content of each sample was determined by using the Bradford assay (Bio-Rad Laboratories, Hercules, CA, USA). A total of 30 μg protein was electrophoresed by using 10% SDS-polyacrylamide gel and then transferred to a polyvinyl difluoride membrane. The membrane was blocked with 5% skim milk for 1 h and incubated overnight at 4 °C with the following primary antibodies: p-65 (diluted 1:1000, Abcam), phosphorylated-p65 (diluted 1:1000, Abcam), iNOS (diluted 1:1000, Abcam), HO-1 (diluted 1:1000, Cell Signaling, Beverly, MA, USA), Nrf2 (diluted 1:1000, Cell Signaling), β-actin (diluted 1:1000, Cell Signaling), lamine B1 (diluted 1:1000, Abcam). After washing three times with tris-buffered saline (TBS) that was supplemented with Tween 20, the horseradish peroxidase (HRP)-conjugated secondary antibody was diluted to 1:3000 and incubated for 1 h. Membranes were washed again, and binding was detected by using an enhanced chemiluminescence kit (Thermo-Scientific, Waltham, MA, USA). The density of each protein band was evaluated by using ChemiDoc (Bio-Rad Laboratories, Hercules, CA, USA).

### 2.8. Statistical Analysis

All data are expressed as mean ± standard deviation. Statistical evaluation was performed by using analysis of variance (ANOVA) followed by Dunnett’s post-hoc adjustments. A value of *p* < 0.05 was determined to be significant.

## 3. Results

### 3.1. Identification of Compounds within SKE by HPLC Analysis

**Figure 2:**
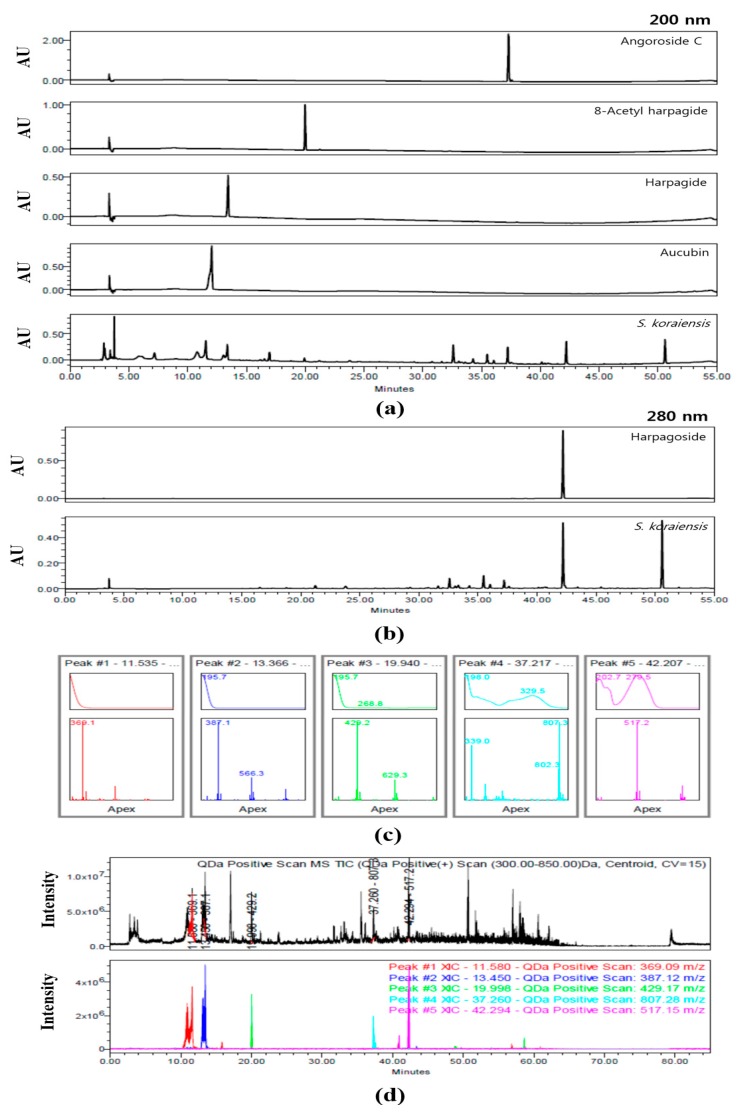
Chromatogram of *S. koraiensis* at 200 nm (**a**) and 280 nm (**b**), UV spectra and MS spectra of peaks according to retention time (**c**), total ion chromatogram (TIC) and extracted ion chromatogram (XIC) (**d**).

The HPLC chromatograms of SKE at 200 and 280 nm are shown in Figure 2. Aucubin (15.70 ± 0.259 μg/mg), harpagide (12.22±0.131 μg/mg), 8-acetyl harpagide (1.64 ± 0.015 μg/mg), angoroside C (2.90 ± 0.014 μg/mg), and harpagoside (6.75 ± 0.049 μg/mg) were detected at approximately 11.5, 13.4, 19.9, 37.2, and 42.2 min, respectively. With the exception of harpagoside, which was detected at 280 nm, the four other compounds were detected at 200 nm (Figure 2a,b). The predominant compounds aucubin and harpagide were classified a iridoids. The five peaks of SKE were also analyzed for their UV wavelength and molecular weight. Their UV spectra were λ_max_ = 195.3, 195.7, 195.7, 198.0, and 279.5 nm. In positive mode, the five compounds were confirmed as follows: aucubin [M+Na^+^]^+^ = 369.1 *m/z*, harpagide [M+Na^+^+OH^-^]^+^ = 387.1 *m/z*, 8-acetyl harpagide [M+Na^+^]^+^ = 429.1 *m/z*, angoroside C [M+Na^+^]^+^ = 807.3 *m/z*, and harpagoside [M+Na^+^]^+^ = 517.2 *m/z* (Figure 2c). The total ion chromatogram (TIC) and extracted ion chromatogram (XIC) were compared to verify the five compounds that were detected in SKE on the chromatogram. In the mass range of 300–850m/z, the TIC was a black line and its XIC values were expressed as follows: Red was aucubin as 369.1 *m/z*, blue was harpagide as 387.1 *m/z*, green was 8-acetyl harpagide as 429.3 *m/z*, cyan was angoroside C as 807.3 *m/z*, and pink was harpagoside as 517.2 *m/z* (Figure 2d).

### 3.2. Effect of SKE on AHR and Inflammatory Cell Count During OVA-Induced Allergic Airway Inflammation

**Figure 3:**
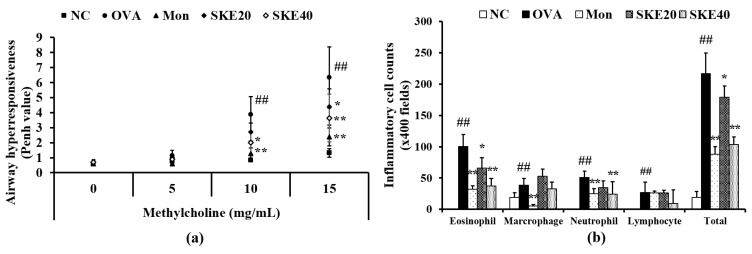
SKE (ethanol extract of *S. koraiensis*) reduced airway hyperresponsiveness (AHR) and inflammatory cell counts during ovalbumin (OVA)-induced allergic airway inflammation. The bronchoalveolar lavage fluid (BALF) was stained with a Diff-Quik agent for cell counting, and AHR was measured by using whole-body plethysmography. (**a**) AHR and (**b**) inflammatory cell counts in the BALF. NC (normal control), PBS (phosphate buffered saline) treatment and PBS sensitization/challenge; OVA, PBS treatment and OVA sensitization/challenge; Mon: montelukast treatment and OVA sensitization/challenge; SKE 20 and 40, SKE treatment (20 and 40 mg/kg, respectively) and OVA sensitization/challenge. Values are shown as the mean ± SD (*n* = 5). ## *p* < 0.01 versus NC; *,** *p* < 0.05 and 0.01 versus OVA.

The OVA-challenged group showed a marked elevation of AHR in comparison to the PBS-challenged controls (Figure 3a). Montelukast treatment significantly reduced AHR compared to the OVA-challenged group that received PBS treatment. The effect of SKE treatment was similar to that of the montelukast treatment, inducing a marked decline of AHR in comparison to that in the OVA-challenged group that received PBS treatment. This reduction was more marked after the high dose (40 mg/kg) SKE treatment.

The OVA challenge led to marked increases in inflammatory cell in the BALF—of eosinophils, in particular (Figure 3b). Both the montelukast treatment and SKE treatments reduced the OVA-induced inflammatory cell increases. The high dose (40 mg/kg) SKE treatment induced the most significant decline in inflammatory cell counts.

### 3.3. Effect of SKE on Pro-Inflammatory Cytokines and IgE Levels During OVA-Induced Allergic Airway Inflammation

**Figure 4:**
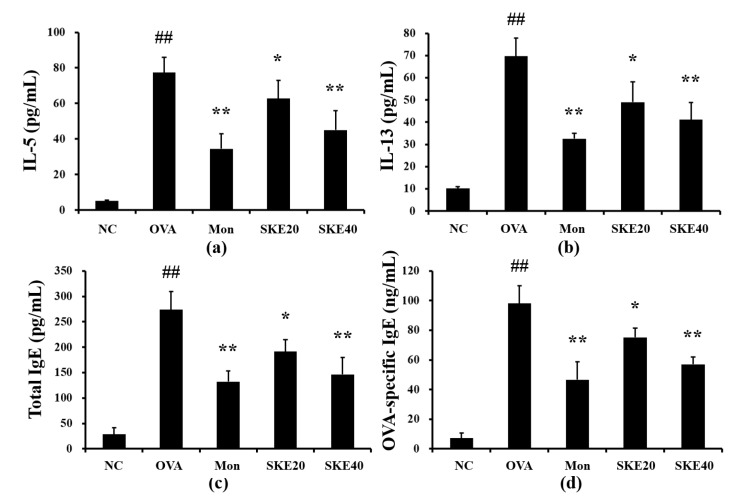
SKE decreased proinflammatory cytokines and immunoglobulin E (IgE) during OVA-induced allergic airway inflammation. Interleukin (IL)-5, IL-13, total IgE, and OVA-specific IgE were determined with commercial ELISA kits. (**a**) IL-5, (**b**) IL-13, (**c**) total IgE, (**d**) OVA-specific IgE. NC, PBS treatment and PBS sensitization/challenge; OVA, PBS treatment and OVA sensitization/challenge; Mon: montelukast treatment and OVA sensitization/challenge; SKE 20 and 40, SKE treatment (20 and 40 mg/kg, respectively) and OVA sensitization/challenge. Values are shown as the mean ± SD (*n* = 5). ## *p* < 0.01 versus NC; *,** *p* < 0.05 and 0.01 versus OVA.

The OVA challenge induced marked elevations of IL-5 and IL-13 in the BALF compared to the PBS-challenged controls (Figure 4a,b, respectively). Both the montelukast and SKE treatments led to a significant reduction in pro-inflammatory cytokines as well as decline in the total IgE and OVA-specific IgE (Figure 4c,d, respectively).

### 3.4. Effect of SKE on Airway Inflammation and Mucus Production During OVA-Induced Allergic Airway Inflammation

**Figure 5:**
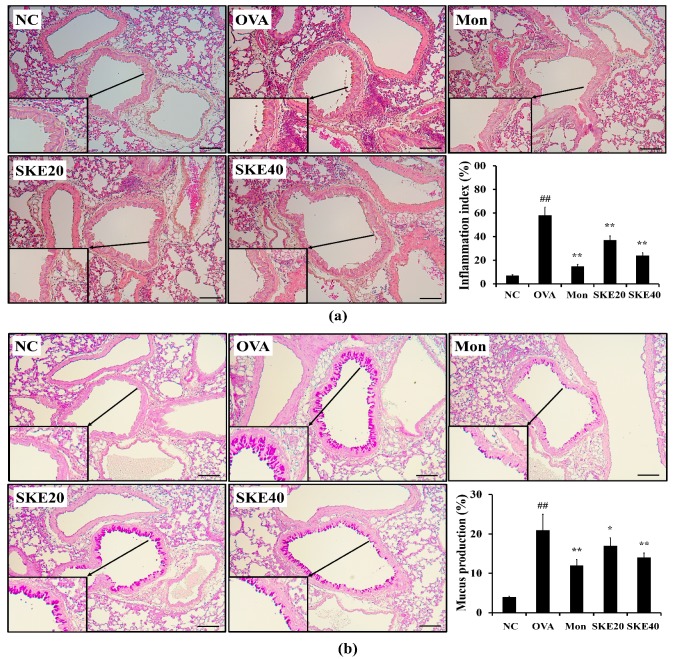
SKE attenuated airway inflammation and mucus production during OVA-induced allergic airway inflammation. Lung tissue samples were stained with hematoxylin and eosin (H and E) and Diff-Quik agents. (**a**) Airway inflammation and (**b**) mucus production. The quantitative analysis of airway inflammation and mucus production were measured by an image analyzer. NC, PBS treatment and PBS sensitization/challenge; OVA, PBS treatment and OVA sensitization/challenge; Mon: montelukast treatment and OVA sensitization/challenge; SKE 20 and 40, SKE treatment (20 and 40 mg/kg, respectively) and OVA sensitization/challenge. Scale bars indicate 50 μm. Values are shown as the mean ± SD (*n* = 5). ## *p* < 0.01 versus NC; *,** *p* < 0.05 and 0.01 versus OVA.

The OVA challenge markedly increased inflammatory cell accumulation in lung tissues compared to the PBS-challenged controls (Figure 5a). Both the montelukast and SKE treatments significantly reduced the OVA-induced inflammatory cell accumulation. Similarly, both treatments also alleviated the increases in the mucus production that was induced by OVA (Figure 5b).

### 3.5. Effect of SKE on NF-κB Phosphorylation and iNOS Expression During OVA-Induced Allergic Airway Inflammation

**Figure 6:**
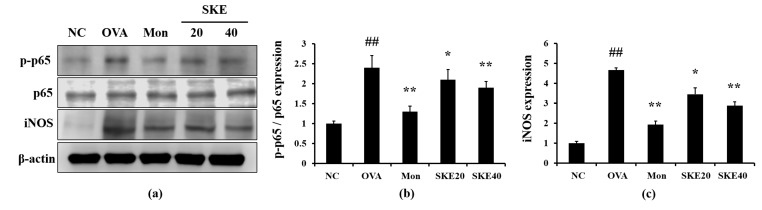
SKE decreased nuclear factor kappa B (NF-κB) phosphorylation and inducible nitric oxide synthase (iNOS) expression during OVA-induced allergic airway inflammation. NF-κB phosphorylation and iNOS expression were measured by western blotting. (**a**) Protein expression on gel, (**b**) relative p-p65/p65 expression value, and (**c**) relative iNOS expression value. NC, PBS treatment and PBS sensitization/challenge; OVA, PBS treatment and OVA sensitization/challenge; Mon: montelukast treatment and OVA sensitization/challenge; SKE 20 and 40, SKE treatment (20 and 40 mg/kg, respectively) and OVA sensitization/challenge. Values are shown as the mean ± SD (*n* = 5). ## *p* < 0.01 versus NC; *,** *p* < 0.05 and 0.01 versus OVA.

**Figure 7:**
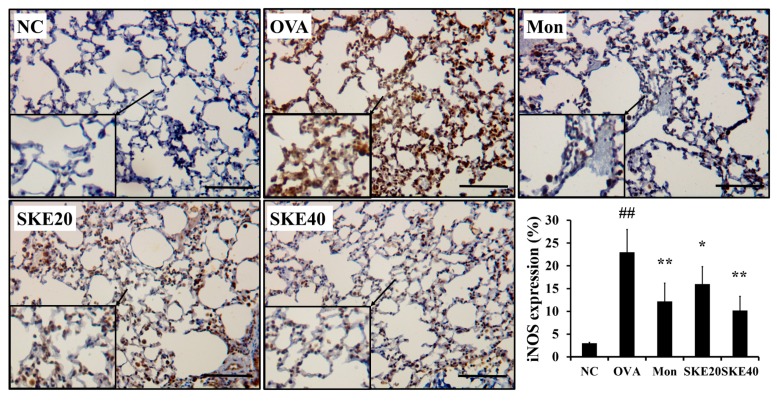
SKE reduced iNOS expression in the lungs during OVA-induced allergic airway inflammation. The expression of iNOS on lung tissue was determined by immunohistochemistry (IHC). NC, PBS treatment and PBS sensitization/challenge; OVA, PBS treatment and OVA sensitization/challenge; Mon: montelukast treatment and OVA sensitization/challenge; SKE 20 and 40, SKE treatment (20 and 40 mg/kg, respectively) and OVA sensitization/challenge. Scale bars indicate 50 μm. Values are shown as the mean ± SD (*n* = 5). ## *p* < 0.01 versus NC; *,** *p* < 0.05 and 0.01 versus OVA.

The OVA challenge led to an increased NF-κB phosphorylation compared to the PBS-challenged controls (Figure 6). Montelukast and SKE treatments reduced the increases in NF-κB phosphorylation. iNOS expression in the lung tissue was also markedly increased in response to the OVA challenge, and this was reduced by SKE treatment, as well as the results of IHC (Figure 7).

### 3.6. Effect of SKE on Nrf2/HO-1 Signaling During OVA-Induced Allergic Airway Inflammation

**Figure 8:**
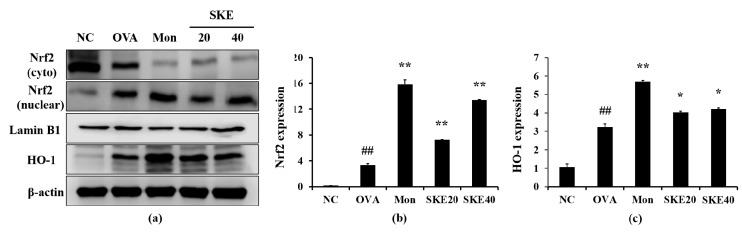
SKE enhanced Nrf2/HO-1 (nuclear factor erythroid-derived 2-related factor/heme oxygenase-1) signaling during the OVA-induced allergic airway inflammation model. The expression of Nrf2 and HO-1 were measured by western blotting. (**a**) Protein expression on gel, (**b**) relative nucleus/cytoplasm of Nrf2 expression value, (**c**) relative HO-1 expression value. NC, PBS treatment and PBS sensitization/challenge; OVA, PBS treatment and OVA sensitization/challenge; Mon: montelukast treatment and OVA sensitization/challenge; SKE 20 and 40, SKE treatment (20 and 40 mg/kg, respectively) and OVA sensitization/challenge. Values are shown as the mean ± SD (*n* = 5). ## *p* < 0.01 versus NC; *,** *p* < 0.05 and 0.01 versus OVA.

**Figure 9:**
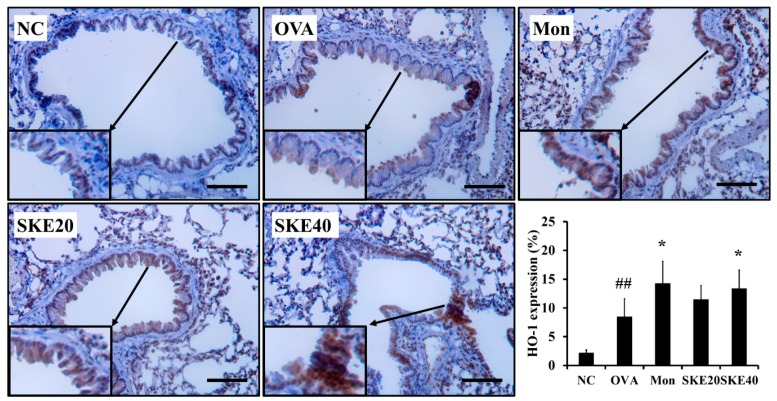
SKE reduced HO-1 expression in the lungs during OVA-induced allergic airway inflammation. The expression of HO-1 on lung tissue was determined by IHC. NC, PBS treatment and PBS sensitization/challenge; OVA, PBS treatment and OVA sensitization/challenge; Mon: montelukast treatment and OVA sensitization/challenge; SKE 20 and 40, SKE treatment (20 and 40 mg/kg, respectively) and OVA sensitization/challenge. Scale bars indicate 50 μm. Values are shown as the mean ± SD (*n* = 5). ## *p* < 0.01 versus NC; * *p* < 0.05 versus OVA.

The OVA challenge increased nuclear Nrf2 expression, whereas the PBS challenge did not (Figure 8a,b). The montelukast and SKE treatments elevated the nuclear Nrf2 expression in comparison to the OVA challenge. Similarly, HO-1 expression was elevated in response to the OVA challenge and more elevated by the SKE treatment (Figure 8a,c). These results were consistent with the results of IHC. The OVA challenge markedly increased the HO-1 expression in lung tissues compared to the PBS-challenged controls, and these increases were greater with the SKE treatment (Figure 9).

## 4. Discussion

Asthma is characterized by the chronic inflammation of the respiratory system, mucus production, and AHR, all of which result in airway obstruction and the interruption of normal airflow [20,21]. In this study, we investigated the protective effects of SKE in an OVA-induced allergic airway inflammation model, focusing on its anti-inflammatory and antioxidant properties. The SKE treatment decreased inflammatory cell counts and reduced IL-5, IL-13, total IgE, and OVA-specific IgE production, together with a reduction in AHR. In addition, SKE reduced NF-κB phosphorylation and iNOS expression in lungs while enhancing Nrf2/HO-1 signaling.

Elevated eosinophil numbers are considered major biomarkers of asthma [22]. Eosinophilia during asthma development is associated with pro-inflammatory cytokines such as IL-4, IL-5 and IL-13 [23,24]. These cytokines are involved in driving the maturation, activation, and accumulation of eosinophils within damaged lung lesions, leading to IgE production, airway inflammation, mucus overproduction, and AHR [25,26]. Therefore, the modulation of pro-inflammatory cytokines is regarded as an important strategy for controlling asthma. In this study, SKE significantly decreased the number of eosinophils during OVA-induced allergic airway inflammation and was accompanied by a reduction in pro-inflammatory cytokines, IgE and AHR. These responses to the SKE treatment were consistent with histopathological examinations that indicated a reduction in airway inflammation and mucus production. These results indicate that SKE has an anti-asthmatic effect.

The phosphorylation of NF-κB plays a major role in allergic responses during allergic asthma. During the development of asthma, phosphorylated NF-κB that is induced by various stimuli translocates to the nucleus and upregulates the expression of various pro-inflammatory genes [27]. In particular, the upregulation of iNOS expression downstream of NF-κB phosphorylation promotes inflammation via the increased production of pro-inflammatory mediators including cytokines, chemokines, and molecules that cause oxidative stress [28,29]. iNOS expression has been shown to be increased and to be accompanied by the upregulation of NF-κB in patients with asthma [30]. During the development of asthma, iNOS expression leads to nitric oxide (NO) production, further exacerbating asthma, as NO is an oxidative stress factor [31,32]. Therefore, the modulation of NF-κB and iNOS signaling is regarded as an important strategy for treating asthma. In this study, SKE significantly reduced iNOS expression and reduced NF-κB phosphorylation during OVA-induced allergic airway inflammation. These results indicate that the anti-asthmatic effects of SKE could be due to modulation of NF-κB phosphorylation and iNOS expression.

Oxidative stress is associated with the development of asthma, and various antioxidative responses are produced to limit oxidative stress-related damage. HO-1 is one of the enzymes that is induced by oxidative stress to act as host defense to reduce oxidative stress and associated inflammatory responses [33]. HO-1 expression is modulated by various transcription factors including Nrf2. Oxidative stress causes Nrf2 to dissociate from Kelch-like epichlorohydrin-associated proteins (Keap 1) and translocate into the nucleus, where it directly binds the antioxidant response elements (AREs) [34]. These events lead to the eventual increase in HO-1 expression, boosting the antioxidant defense system [35]. Many studies have also demonstrated that Nrf2/HO-1 signaling is involved in inflammatory responses. The upregulation of Nrf2/HO-1 signaling suppresses inflammatory responses via the suppression of NF-κB signaling in a number of inflammatory diseases [33,36]. In this study, SKE induced the nuclear translocation of Nrf2 during OVA-induced allergic airway inflammation, which resulted in the overexpression of HO-1. These results indicate that SKE could attenuate asthmatic response via the activation of Nrf2/HO-1 signaling. The antioxidant effect of SKE has also been supported by previous studies [37,38]. In other members of the *Scrophularia* genus, *Scrophularia umbrosa* has shown strong free radical scavenging activity [37], and *S. buergeriana* has been shown to enhance the Nrf2/HO-1 signaling pathway [38].

In this study, SKE showed an anti-asthmatic effect on an OVA-induced asthma model. However, the effects of SKE are somewhat smaller than the effects of montelukast. Montelukast, a leukotriene receptor antagonist, is clinically used to relieve allergic airway inflammation [39]. The difference between SKE and montelukast is considered to be due to the chemical composition of each material. In this study, montelukast was used as a single compound, but SKE was used as an extract that consisted of various compounds. Therefore, further studies are needed to find the active single compound of SKE and investigate its efficacy for asthma.

## 5. Conclusions

In conclusion, SKE reduced eosinophilia and pro-inflammatory cytokines production, decreased IgE levels, and alleviated AHR during OVA-induced allergic airway inflammation, as supported by histological evidence of reductions in airway inflammation and mucus secretion. These anti-asthmatic effects of SKE were closely associated with the suppression of NF-κB phosphorylation and the activation of Nrf2/HO-1 signaling. Our results suggest that SKE may be an effective therapeutic for suppressing asthma development.

## Supplementary Materials

The following are available online at https://www.mdpi.com/2076-3921/9/2/99/s1, Figure S1: Stereomicroscope micrographs showing the external morphology of Scrophularia koraiensis Nakai., Table S1: Comparison of morphological characteristics in two species in Scrophularia.
