# Supplementary material for: Scrophularia koraiensis Nakai Attenuates Allergic Airway Inflammation via Suppression of NF-κB and Enhancement of Nrf2/HO-1 Signaling
Source: Antioxidants (Basel). 2020 Jan 24;9(2):99. doi: 10.3390/antiox9020099 (PMC7070852; doi:10.3390/antiox9020099)
Supplement: Supplementary file 1 [file antioxidants-09-00099-s001.pdf]

antioxidants

MDPI

Table S1. Comparison of morphological characteristics in two species in *Scrophularia*.

	Scrophularia buergeriana	*Scrophularia koraiensis*
Apex of leaf blade	acute	acuminate
Base of leaf blade	usually truncate	usually obtuse
Margin of leaf blade	regularly double serrate	regularly double serrate
Calyx lobes	ovate	acuminate

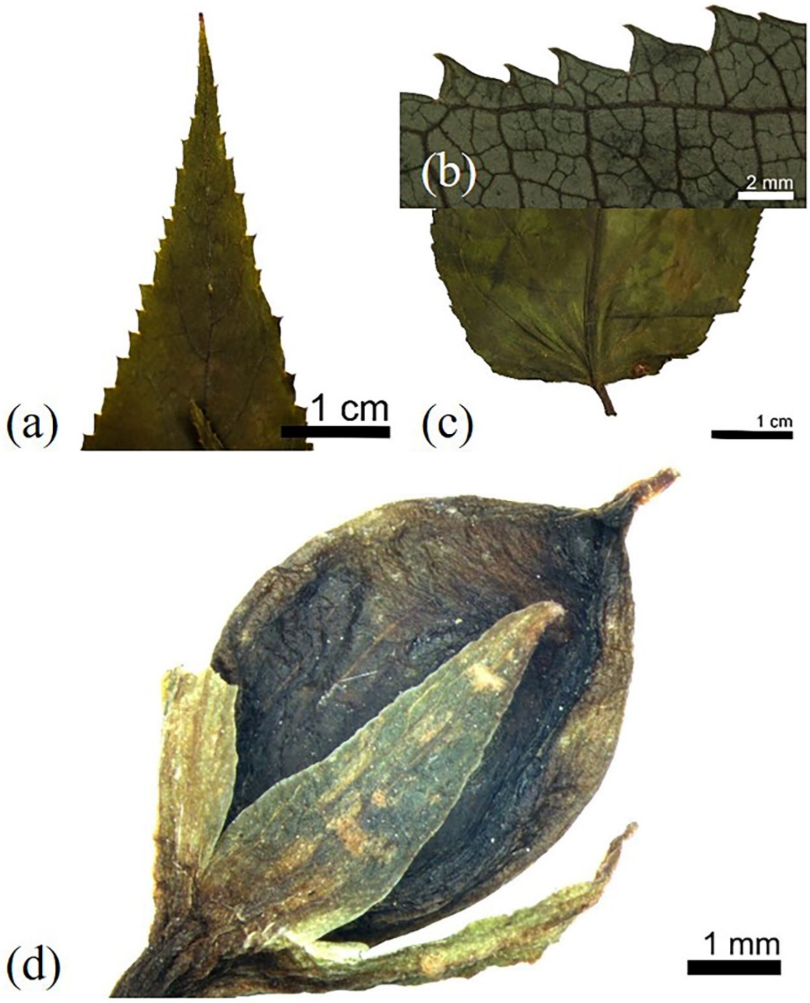

Figure S1. Stereomicroscope micrographs showing the external morphology of *Scrophularia koraiensis* Nakai. (**a**) Apex of leaf blade. (**b**) Margin of leaf blade. (**c**) Base of leaf blade. (**d**) Fruit and calyx.
